# Comparative Pathobiology of Canine and Human Prostate Cancer: State of the Art and Future Directions

**DOI:** 10.3390/cancers14112727

**Published:** 2022-05-31

**Authors:** Eduardo de Paula Nascente, Renée Laufer Amorim, Carlos Eduardo Fonseca-Alves, Veridiana Maria Brianezi Dignani de Moura

**Affiliations:** 1 School of Veterinary Medicine and Animal Science, Federal University of Goiás, Goiânia 74001-970, Brazil; eduardonascente@discente.ufg.br; 2 Veterinary Clinic Department, School of Veterinary Medicine and Animal Science, São Paulo State University (UNESP), Botucatu 18618-970, Brazil; renee.laufer-amorim@unesp.br; 3 Department of Veterinary Surgery and Anesthesiology, School of Veterinary Medicine and Animal Science, São Paulo State University (UNESP), Botucatu 18618-970, Brazil; carlos.e.alves@unesp.br

**Keywords:** Immunobiology, molecular biology, oncogenes, prostate carcinoma, tumor suppression

## Abstract

**Simple Summary:**

Prostate cancer (PC) is one of the main types of cancer that affects the male population worldwide. In recent decades, there has been a significant evolution in the methods of diagnosis and treatment, mainly due to the development of new research in the field of molecular biology, allowing for a better understanding of how this cancer develops and progresses from a genetic point of view. In addition to humans, the canine is the only mammal that develops PC spontaneously. However, in dogs, PC has a distinct form, with high lethality rates. This work of scientific characterization of cancer in humans and dogs approaches how differences and similarities already all explain how PC develops and progresses in dogs, with a central tenet of molecular biology, that is, the transport between DNA, RNA and proteins. The understanding of these mechanisms makes it possible to identify new methods of the diagnosis and treatment of the disease, not only in men, but also in dogs.

**Abstract:**

First described in 1817, prostate cancer is considered a complex neoplastic entity, and one of the main causes of death in men in the western world. In dogs, prostatic carcinoma (PC) exhibits undifferentiated morphology with different phenotypes, is hormonally independent of aggressive character, and has high rates of metastasis to different organs. Although in humans, the risk factors for tumor development are known, in dogs, this scenario is still unclear, especially regarding castration. Therefore, with the advent of molecular biology, studies were and are carried out with the aim of identifying the main molecular mechanisms and signaling pathways involved in the carcinogenesis and progression of canine PC, aiming to identify potential biomarkers for diagnosis, prognosis, and targeted treatment. However, there are extensive gaps to be filled, especially when considering the dog as experimental model for the study of this neoplasm in humans. Thus, due to the complexity of the subject, the objective of this review is to present the main pathobiological aspects of canine PC from a comparative point of view to the same neoplasm in the human species, addressing the historical context and current understanding in the scientific field.

## 1. Introduction

Cancer is considered a leading cause of death in humans before the age of 70 years in 112 of 183 countries, with a significant increase in incidence and mortality rates in recent years [1]. According to the Global Cancer Statistics (Globocan), prostate cancer is the third most frequent neoplastic entity, following breast and lung cancer [2]. It is the main neoplasm diagnosed in men in more than half of the countries in the world, with a high incidence in developed ones, with 1,414,259 cases being diagnosed worldwide in 2020, with 375,304 deaths [2,3].

Prostate cancer (PC) in dogs is a neoplasm of undifferentiated morphology—aggressive and with high rates of metastasis to regional lymph nodes, lungs, liver, and, mainly, bones [4]. Unlike humans, PC is commonly diagnosed in dogs at advanced stages, and patients have a short survival period and poor quality of life. This scenario has been justified by the ineffectiveness of androgen-dependent screening and diagnostic tests, such as growth factors, as well as the absence of effective treatment protocols [5].

Oncogenes and tumor suppressor genes have dominated the basic scientific research of tumorigenesis with recent advances in molecular biology, so that their evaluation and protein products may provide new diagnostic biological markers [6]. In addition, investigation and understanding of the molecular pathways of carcinogenesis involved in a prostate disease can add data to clinical information, helping to predict tumor progression at an early stage of cancer and identify new responsive therapeutic targets, as the treatment for PC in the canine species is still ineffective [7].

Gaps still need to be filled, although science has progressed significantly in recent decades to elucidate the molecular mechanisms involved in the carcinogenesis and progression of canine PC. Due to the complexity of the subject, this review aims to present the main pathobiological aspects of canine PC from a comparative point of view, addressing the historical context and current understanding in the scientific field.

## 2. History and Epidemiological Aspects of Prostate Cancer

It is possible to identify that the first records of prostate cancer date from the 19th century. Based on scientific publications, in 1817, the English surgeon and pathologist George Langstaff described, for the first time, prostate cancer in men from an anatomo-macroscopic perspective [8]. His article published in the Medico-Chirurgical Transactions, entitled “Fungus Haematodes,” describes the case of a 68-year-old man with symptoms of abdominal pain and hematuria, besides urethral obstruction and prostate enlargement on rectal examination. However, only on autopsy did the pathologist confirm that it was a prostate neoformation extending into the urethra and bladder [8].

Other cases of PC were published in the scientific communities of Germany and France until the year 1850, but it was still considered by many an uncommon disease. However, in 1853, the physician J. Adams, an experienced microscopist at *The London Hospital*, described the first case of metastatic prostate cancer established by histopathological examination [9]. Then, researchers reviewed cases around the world for years, trying to better understand the prostate and its diseases. However, it was only at the turn of the last century, when Albarran and Halle [10] started histopathological evaluations of several samples of this gland, that malignant changes in the prostate tissue were really identified and recognized.

One of the milestones in understanding the pathology, epidemiology, and therapeutic aspects of prostate cancer occurred in 1896, when Reginald Harrison stated that this neoplasm was similar to hypertrophic lesions, in addition to having a higher incidence than was believed at that time [11]. This researcher had carried out, in 1884, one of the first surgical–therapeutic approaches aimed at prostate cancer [12], and his discoveries opened new paths for investigating the efficiency of other surgical methods, such as radical prostatectomy [13] and vasectomy [14].

Other studies seeking therapeutic alternatives were conducted still with conflicting results about surgical methods, such as the use of radioactive isotopes [15]. The response of sex hormones and their relationship with the prostate were also widely investigated at the same time that the canine species began to be used as one of the first experimental models for the study of prostatic diseases in men. In this context, initially in 1893, J. William White Jr. [16] observed that dog castration promoted the atrophy of glandular elements, followed by a reduction in the prostate volume. Despite this, it was only in 1939 that Huggins and collaborators [17,18] investigated the relationship between castration associated with estrogen administration and the blockade of prostatic secretions and cell atrophy in elderly dogs.

Around 80 years later, there has been a significant increase in the incidence and mortality by PC in men worldwide, affecting approximately 1.5 million patients [3]. Although variable, high prevalence has been observed in countries such as Australia and Japan, as well as in North America and Western Europe. However, the highest mortality numbers were identified in underdeveloped countries, such as some from Africa, and Central and South America. This scenario may be explained by some risk factors related to socioeconomic and racial aspects, although complex, aging-related risk factors, genetic factors such as BRAF mutation, race (black men are more predisposed), and limited access to diagnostic tests and therapeutic protocols could justify these differences [2,3].

Age is associated with higher occurrence and mortality rates, and men over 65 years of age are more susceptible to developing PC [19,20]. In a comprehensive review, Rawla [19] addresses risk factors associated with the development of prostate cancer in men, including racial and ethnic factors, as black and African-American men have the highest incidence and chances of developing PC earlier. Genetics, family history, type of diet, mineral and vitamin deficiencies, alcohol consumption, obesity, hyperglycemia, and environmental exposure to chemicals or radiation are also considered important risk factors for PC development [19].

Over time, dogs have still been considered the main experimental model to help understand the tumor biology of PC since the first study carried out using these animals in experiments, defining molecular bases and investigating new efficient therapeutic methods. Initial investigations in the 1900s report and describe morphologically the PC in dogs [21], proving that canine is the only mammalian species that spontaneously develops PC more often than man and exhibits similar histological characteristics that allow the assessment of tumor development and progression [22]. Furthermore, even in the face of alternatives, such as the use of transgenic mice and xenotransplantation [23], it is known that these models do not simulate the characteristics and complexity of the disease in humans [24].

The prevalence of spontaneous PC in dogs is low when compared to men, being recorded between 2–12% and varying significantly according to the adopted number of samples and experimental design [25]. Teske et al. [26] and Polisca et al. [25] evaluated canine prostate disorders in a large sample and found prostate tumors in 12.99 and 2.6% of cases, respectively. However, a retrospective analysis of canine PC revealed an incidence of less than 1% [27]. Moreover, the prevalence and mortality rate of PC in dogs is strongly related to increasing age, being more frequent in dogs over seven years old [26,27].

However, questions need to be clarified for the canine species, such as castration, an approach that is still questionable and widely discussed by the veterinary scientific community. Although castration has been considered for years to be the most appropriate therapeutic method for prostate disorders, the role of this approach as a risk factor for the development of canine PC has been studied in the last two decades [27]. According to researchers, adult and castrated male dogs are more likely to develop PC [25,26], whereas for others authors, the castration procedure does not reflect much on tumor progression or reduced chances of developing PC [28,29]. Despite efforts, there is still not enough evidence to support these theories, as experimental limitations prevent a consistent assessment, such as the size of the reference population, when the patient was castrated throughout life before the neoplasm was diagnosed. [27].

## 3. Preneoplastic Prostatic Lesions

**Figure 1:**
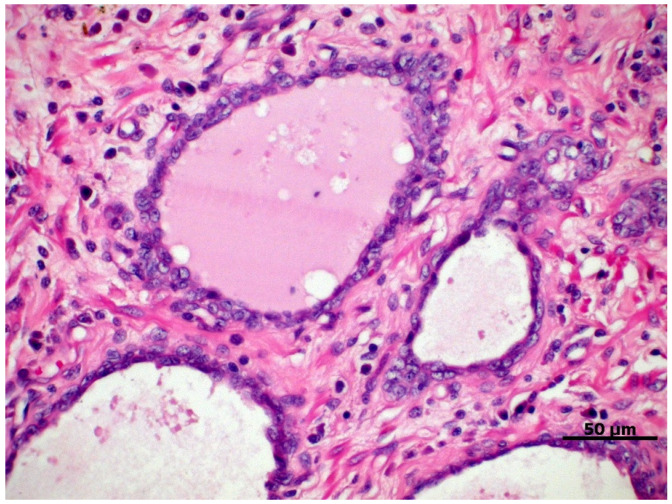
Proliferative inflammatory atrophy in canine prostate, characterized by discrete proliferation of prostatic epithelium associated with mononuclear inflammatory infiltrate in the glandular interstitium. HE, 40×.

A couple of dysplastic lesions (Figure 1) are considered preneoplastic and have been studied for a better understanding of prostate carcinogenesis due to their potential for progression to PC, either because of their histomorphological similarity or because they exhibit potential carcinogenic molecular factors [30,31]. Two main lesions are recognized in the human prostate, that is, the proliferative inflammatory atrophy (PIA) and prostatic intraepithelial neoplasia (PIN), also described in the canine prostate [30,31].

PIA is characterized by dysplastic, atrophic, and/or proliferative changes in the prostatic epithelium, associated with varying degrees of mononuclear inflammatory infiltrate in the glandular interstitium (Figure 1) [32]. In dogs, it is a commonly diagnosed prostate disorder often associated with prostatic hyperplasia, intraepithelial neoplasia, and PC, which can be found from the vicinity of the urethra to the pericapsular prostatic parenchyma of the medial portion of the prostate [31].

Lesions related to PIA in the canine species have a marked proliferative potential when compared to normal prostate tissue, with a higher population of intermediate cells. These findings suggest that this condition originates from the proliferation of basal cells stimulated by the continuous inflammatory process [33], corroborating the findings by Palmieri et al. [34], who report marked immunostaining of cytokeratin-5 (CK5) in many of the cuboidal luminal epithelial cells. These characteristics are phenotypically similar to some prostatic carcinomatous lesions in dogs, thus implying that most of these cases may have a similar origin [35].

Genotypically, changes related to p53 protein expression can be initially observed in PIA and accentuated in canine PC since the overexpression of this protein is associated with higher proliferative indices [36]. Furthermore, PIA samples also show down-regulation of phosphatase and tensin homolog (PTEN), the androgen receptor gene, and its protein levels [33]. These changes in gene expression are important in prostate carcinogenesis, considering that the down-regulation of PTEN is involved in the activation of anti-apoptotic pathways and strongly correlated with loss of androgen receptors, a common event in PC [37,38].

PIN is considered the main precursor lesion of prostate cancer in men, mainly high-grade, and often diagnosed as a lesion adjacent to prostate adenocarcinoma [39,40]. On the other hand, PIN has a low occurrence in intact dogs and, when present, tends to present as a focal lesion [41]. Its actual incidence is still considered questionable and probably overestimated or not correctly diagnosed in many studies, besides its role in canine PC carcinogenesis still to be discussed [33].

The first reports of high-grade PIN in sexually intact dogs were published in the 1990s [42,43], in which cyto-histomorphological changes similar to those observed in the corresponding lesions in the human prostate are described. Morphologically, PIN shows foci of the proliferation of pre-existing ducts and acini with nuclear stratification and cell agglomeration, mild to moderate cell pleomorphism, anisokaryosis, and atypical basal cells [44].

However, under investigation, PIN characterization studies have been carried out to verify its potential in the carcinogenesis of canine PC. Despite exhibiting heterogeneous labeling for androgen receptors, basal cells in these lesions are believed to act in prostate tumor development with significant potential for proliferation [44]. Moreover, PIN exhibits an immunophenotype similar to PIA and canine PC with regard to cell cycle regulators [36], showing lower expression of TGF-β in epithelial cells and tissue stroma, referring to the impossibility of this cytokine to inhibit cell proliferation [45].

Palmieri et al. [46] suggested the role of heat shock protein 90 (HSP90), a chaperone protein, in canine prostate carcinogenesis and tumor progression and detected increased immunohistochemical labeling of this protein in PIA and PIN lesions, especially in the nuclear site. Assuming that HSP90 acts to promote essential metabolic pathways of tumorigenesis through its functions as a molecular chaperone protein, stimulating cell proliferation, its high expression in these lesions can be interpreted as an early and important event in the development of canine PC [47].

In fact, the detection of high-grade PIN in humans has clinical relevance, and its differentiation from other benign and intraductal lesions is still considered a challenge, mainly attributed to interobserver variability [48]. Thus, after high expression in cases of human PC being reported [49], the cyclin-dependent kinase 19 (CDK19) was identified as a specific and sensitive biomarker for the diagnosis of high-grade PIN, reflecting its involvement in the progression of neoplastic disease, as well as its malignant potential [50]. However, investigations of this molecule still need to be registered in canine prostate samples, and it is still necessary to identify and compare the signaling pathways that play an essential role in canine PIN development [51].

## 4. Classification and Histological Grading of PC

Histologically, canine prostate cancer exhibits conflicting differentiation. Initially, the classification adopted by the veterinary community was based on that recommended by the World Health Organization, which includes two main types of prostate cancer, i.e., adenocarcinoma, with intra-alveolar and acinar subtypes, and poorly differentiated carcinoma [52]. Over time, there was a need to create a consensus in veterinary pathology aiming to assist pathologists in the process of classification and grading of these lesions, considering the histological variability reported in the literature.

Therefore, in 2019, Palmieri et al. [53] presented the histopathological terminology standards for epithelial lesions of the prostate in dogs, intending to provide veterinary pathologists with basic, comprehensive, and practical atlas. In addition to describing recognized lesions such as hyperplasia, atrophy, inflammatory processes, and non-invasive proliferative epithelial lesions of the prostate, the researchers point out that three types of prostate carcinoma can be diagnosed through histopathology, that is, urothelial carcinoma of the prostate (UCP), prostate adenocarcinoma (PA), and mixed prostatic carcinoma (MPC), with urothelial and glandular phenotypes [53].

**Figure 2:**
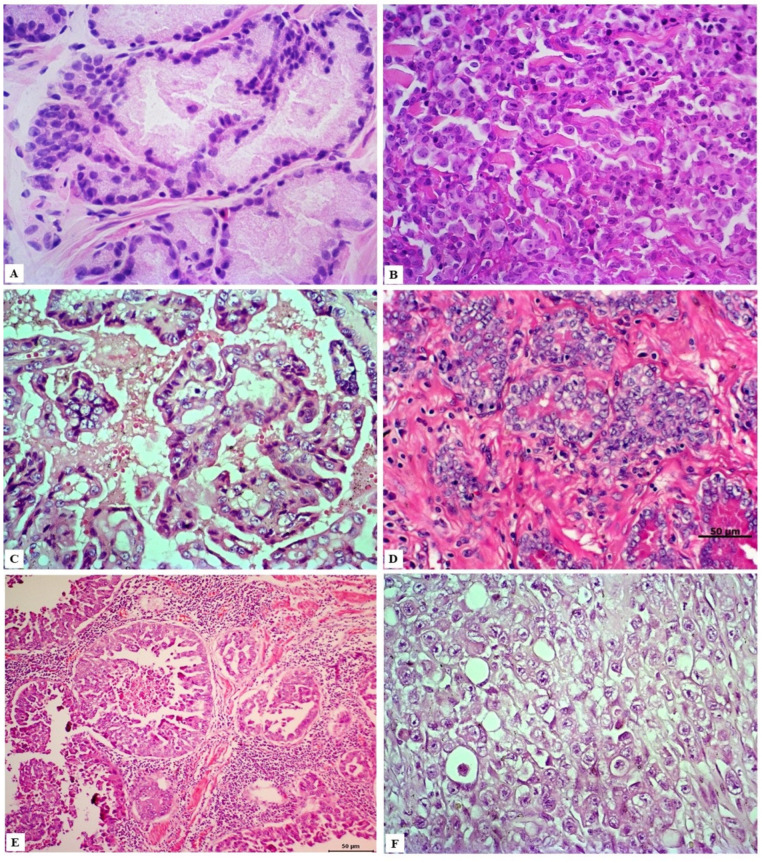
Histological patterns of canine PC. (**A**) Normal prostate. HE, 200×. (**B**) Solid/undifferentiated, showing pleomorphic cells with no specific growth pattern. HE, 200×. (**C**) Papillary, represented by neoplastic ductal epithelial cells exhibiting tubulopapillary projections. HE, 200×. (**D**) Small acinar streaky by dense fibrovascular stroma. HE, 50 µm. (**E**) Cribriform, characterized by neoplastic ductal cells forming irregular fenestrae with a central area of necrosis. HE, 50 µm. (**F**) Signet ring cells characterized by intracytoplasmic vacuolization with nuclear displacement to the periphery. HE, 200×.

However, different morphological subtypes have been described in the literature due to the high degree of histological heterogeneity (Figure 2) and, therefore, papillary, solid/undifferentiated, cribriform, mucinous, signet-ring, and acinar/ductal patterns should also be considered in the diagnosis of prostate adenocarcinoma [54,55,56,57,58]. Importantly, these histological patterns are also diagnosed in the human prostate, as well as additional lesions, including mucinous fibroplasia, squamous metaplasia, and perineural and lymphatic invasions, which are observed in both species [54,58,59].

Prostate carcinoma in humans is graded based on the Gleason system, an important prognostic indicator [60]. This system, created in 1966 by Donald F. Gleason, assigned grades from one to five to the microscopic architectural pattern of the tumor, being patterns from one to three referred to well-differentiated neoplastic lesions, whereas patterns four and five exhibited markedly abnormal glandular architecture [60]. However, the discovery of new screening techniques, biopsy protocols, and the identification of new growth patterns or PP variants over the following years [61] led this system to undergo significant changes in 2005 and 2014, mediated by the International Society of Urological Pathology (ISUP) [62,63].

The modified Gleason system (GS) was designed in such a way that prostate carcinoma is classified based on the assignment of scores/grades to present primary and secondary lesions, and the sum of both are classified in a certain group, namely: grade 1 group (GS ≤ 6), grade 2 group (GS 3 + 4), grade 3 group (GS 4 + 3), grade 4 group (GS 8), and grade 5 group (GS 9–10) [62,63]. Thus, the review of the Gleason system resulted in a more objective classification among the examiners, providing greater prognostic certainty for patients [64,65,66].

In this scenario, and based on the numerous similarities between human and canine PC, Palmieri and Grieco [67] applied the modified Gleason system to classify prostate carcinomas in dogs. The highest score (Gleason 10) was attributed in almost half of the cases after analysis of samples from biopsy, necropsy, and prostatectomy, mainly in patients with metastatic disease. Furthermore, most samples had secondary and tertiary growth patterns, a characteristic similar to that described in human disease. Therefore, the modified Gleason system could be applied in the practice of veterinary pathology to help the oncologist better manage the canine oncological patient [67].

The modified Gleason system can be critically influenced because it is still a subjective assessment, despite having an interobserver reproducibility above 80% [68], requiring the adoption of safer methods. Aiming to eliminate this problem, the analysis of nuclear morphometry presents itself as an auxiliary tool in the classification of cases of canine PC in an agile and safe way regarding the degree of cell differentiation, assisting in determining the survival rate of affected patients [69]. In fact, studies are still needed for validation and addition of cytohistological aspects, but it is clear that this type of analysis has high prognostic potential [69].

## 5. Cell Origin and Tumor Initiation

**Figure 3:**
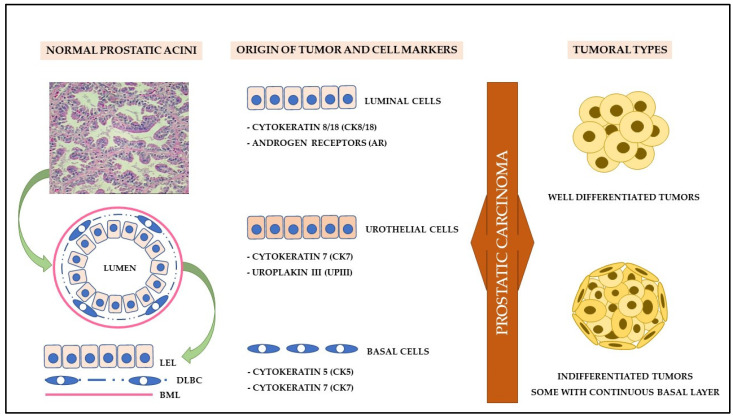
Cellular origin and tumor initiation of the canine PC. The normal prostate of the dog has prominent acini with a smaller stromal component and a discontinuous basal cell layer (DLBC). Thus, canine PC can originate from luminal epithelium layer (LEL), with immunoexpression for low molecular weight cytokeratins (CK8/18) and AR; or originate from basal cells, with immunostaining for high molecular weight cytokeratins (CK5 and CK7), which is more frequent in tumors with a lower degree of cell differentiation, which may also have a continuous basement membrane. In addition to the basal and luminal cells of the prostatic acini, the canine PC may originate from urothelial cells present in the prostatic ducts that empty into the urethral canal, which express CK7 and UPIII. BML: Basement membrane layer.

Although dogs and men share several similarities in prostate disorders, some histomorphological differences act as essential factors for understanding PC carcinogenesis. In dogs, the prostate has a uniform morphology, not being divided into zones, with more prominent acinar structures and a smaller stromal component [70]. Furthermore, unlike humans, healthy canine prostate tissue exhibits a discontinuous basal cell layer, with a smaller cell population in acini, strong nuclear staining for p63 and AR, suggesting the involvement of basal stem cells in the development of canine PC (Figure 3) [71].

This same morphological and immunohistochemical pattern was also reported by Fonseca-Alves et al. [72]. However, when reporting the aberrant expression of p63 in canine PC samples, the authors found a continuous layer of basal cells in neoplastic lesions, accompanied by cytokeratin immunostaining of high (basal cell markers) and low molecular weight (luminal cell markers), differing from the immunoexpression pattern of human PC. In these cases, PC is believed to originate from luminal cells and presents immunoexpression of basal cell markers according to dedifferentiation or vice versa, being able to develop from basal cells and differentiate into luminal cells [72].

Akter et al. [35] demonstrated that the varied growth patterns of canine PC suggest different tumor-initiating cell models. In this context, well-differentiated luminal cells were predominant in canine prostate tumors since most of them are capable of expressing AR and CK8/18. According to the authors, solid PC seems to exhibit an undifferentiated or aberrant phenotype among the histological subtypes, with higher expression of CK14 and significant loss of AR immunoexpression. However, the role of basal cells must be considered important in carcinogenesis, as well as the existence of intermediary cells as an alternative pathway for the neoplastic transformation of the prostate [35].

In addition to basal and luminal markers, canine PC can also be derived from prostatic ducts and not only from acinar cells, considering that a significant portion of PC samples express uroplakin III (UPIII), a transmembrane protein expressed by apical urothelial cells [57,73,74]. Together with CK7, UPIII was considered a marker of significant specificity for urothelial differentiation, that is, for the diagnosis of canine urothelial carcinoma of the prostate since the expression of p63 and high molecular weight cytokeratin is scarce or absent in these cases [57,73,74]. However, immunostaining for UPIII may be mild or negative in some tumors with a mixed histological pattern [74].

## 6. Cancer Stem Cells (CSC)

Cancer stem cells (CSC) significantly contribute to PC carcinogenesis because they remodel the tumor microenvironment, being responsible for maintaining the capacity for cell proliferation, survival, and motility [75,76], besides resisting apoptosis and chemotherapy. These cells are mainly responsible for attributing the phenotypic and functional heterogeneity of several tumors, as they exhibit the property of cell self-renewal [77]. Although limited, the investigation of CSC markers has already been carried out in samples of canine prostate cancer [77,78].

Despite the progress in elucidating the cellular origin of canine PC, CSC may exhibit a similar character to stem cells, an aspect considered important in the tumor initiation process [77]. Canine PC has a complex expression pattern for several stem cell markers, with prominent immunoexpression for CD44, CD133, ITGA6, and DDX5, stem cell markers known to be involved in human PC cases [78]. In addition, most of these markers can be used as a predictor of cell dedifferentiation and, consequently, tumor progression, as CSC expand in the symmetrical division and excessively increase cell growth, thus contributing to recurrence, metastasis, tumor heterogeneity, multidrug resistance, and resistance to traditional therapeutic methods [79].

Costa et al. [77] showed, for the first time, a strong correlation between CSC markers (OCT3/4, CD44+/CD24−, NANOG, and Nestin) and the properties of stem cells in samples and cell lines of canine PC. Among the evaluated markers, cells with the CD44+/CD24− phenotype were found only in tumor samples, unlike the others, which exhibit different degrees of immunostaining in normal and hyperplastic prostate tissues. Immunoexpression of the CD44+/CD24− phenotype was directly correlated to PC cases with higher Gleason scores and shorter patient survival times, reflecting a proliferative, tumorigenic, metastatic, and resistant to chemotherapy and in vivo radiotherapy phenotype [77,80,81].

Other proteins, such as Sox9 and survivin, also play an important role in cell proliferation and can be considered stemness markers in canine prostate cells [82]. However, basal layer cells tend to present strong survivin nuclear immunostaining, which allows us to infer that they are amplifying cells that maintain different stem cell properties [82]. As for survivin, Hood et al. [83] showed that canine PC exhibits strong immunostaining for Ephrin-A3 (EphA3), a receptor tyrosine kinase expressed by the CSC and mesenchymal stem cells of tumor stroma, which is related to more aggressive tumor behavior [84].

## 7. Molecular Biology of Prostate Cancer

Cell populations face obstacles and molecular mechanisms during cancer progression that restrict or favor their development. To investigate potential therapeutic targets aimed at controlling the progression of prostate cancer, it is necessary to better understand the involved biomolecular evolutionary mechanisms, such as the role of androgen signaling in carcinogenesis, the signaling pathways and proliferative markers, the correlation between oncogenes and mechanisms of tumor suppression, cell adhesion and epithelial-mesenchymal transition, and the tumor microenvironment modulation [85,86].

### 7.1. Androgenic Signaling: A Key Point in Prostate Carcinogenesis?

Androgens play a key role in the development and maintenance of male reproductive physiology, with the testes and adrenal glands being responsible for the steroid hormones production as testosterone and dihydrotestosterone, respectively. These hormones physiologically bind to androgen receptors (AR) [87]. AR regulate gene expression with several functions, including protein secretion, gene fusion, and stimulation of cell growth, growth factors, and metabolic enzymes, in addition to acting in cell cycle regulation and glucuronidation [88].

Since the 1940s, Huggins and Hodges [89] have shown that surgical castration in men with prostate cancer significantly improves symptoms, in addition to promoting tumor regression. Since then, experimental studies have shown that the androgen signaling axis has a fundamental role in the pathogenesis of human PC, allowing therapies based on androgen deprivation to become first-line therapeutic protocols for most affected patients [90]. However, resistance to combined hormone deprivation therapy and recurrent cases with metastasis even after gonadectomy began to be observed, with these tumors being called castration-resistant prostate cancer (CRPC), which are characteristically more aggressive [91].

AR are directly related to the onset and progression of prostate tumors in men, with several underlying mechanisms involved, especially in those patients with CRPC, including amplification or mutations in receptors, changes in androgen biosynthesis, and/or changes in the binding cofactor of AR, resulting in modification of its transcriptional activity [92]. However, insights into human PC tumor biology show that many patients with advanced disease may exhibit molecular changes in pathways unrelated to AR, such as BRCA1 and BRCA2 mutations, genes involved in DNA repair (BRCA1/2) [93].

In contrast, canine PC is presented as an androgen-independent neoplasm. This scenario is often justified by the fact that the canine disease exhibits more aggressive behavior, with losses in AR expression, without evident mutations in its DNA-binding domain, making it clinically similar to hormone-resistant PC cases in man [94,95]. However, these characteristics retake the idea that castrated animals may have a greater chance of developing PC, corroborating the findings of Lai et al. [94], who detected partial loss of nuclear AR expression and occurrence of an expression in the cytoplasm of prostatic epithelial cells after castration [96].

The comparative genomic hybridization technique allowed the observation that the canine PC negative for AR is a complex neoplastic entity, characterized by marked genetic instability [4]. Aberrations in the number of DNA copies have been identified on almost all chromosomes, except for chromosome 19, as well as the identification of 655 cancer-related genes, with different signaling pathways and molecular interaction networks. Furthermore, the interspecies cross-validation analysis (man versus dog) showed 79 genes altered by genomic losses in both species, evidencing molecular similarities between human and canine PC [4].

In addition to the loss of AR protein expression in canine PC cells, the same scenario was detected for estrogen receptor alpha (REα) and PTEN [96]. These findings denote an immunohistochemical phenotype similar to PC resistant to hormone therapy in humans, a similarity also reinforced by Kato et al. [97] and Azakami et al. [98]. According to these authors, PC cells may exhibit high levels of expression of the small glutamine-rich tetratricopeptide repeat-containing protein alpha (SGTA), a molecule involved in the inhibition of AR signaling in androgen-independent human and canine prostate cancer cells.

Another aspect involved in AR signaling and considered a risk factor for the development and aggressiveness of human prostate cancer refers to the number of polyglutamine (polyQ) repeats in the N-terminal transactivation domain of the AR gene [99]. Its length is inversely correlated with vigorous stimulation of genes involved in early oncogenic transformation, accelerated tumor cell growth, and aggressive PC tumor phenotypes [100]. Recently, this correlation was detected in dogs, in which shorter polyQ mutants were associated with increased AR signaling activity [101]. However, considering that the polyQ tract polymorphism exhibits population heterogeneity in humans, further studies are needed to better elucidate the underlying pathophysiological mechanisms in the canine species [101].

### 7.2. mTOR/4E-BP1/eIF4E Cell Signaling Pathway

Activation of the phosphatidylinositol 3-kinase (PI3K), protein kinase B (PKB-AKT), and mammalian target of rapamycin (mTOR) signaling pathway, known as PI3K/PKB-AKT/mTOR, is involved in multiple cell-signaling cascades related to tumor survival, initiation, and progression, being also able to decrease the sensitivity of PC cells to targeted therapies [102]. The activation of mTOR, a serine-threonine kinase, leads to the phosphorylation of eIF4E-binding protein 1 (4E-BP1), which promotes the release of eukaryotic initiation factor (eIF4F), which, in turn, allows for the translation of specific mRNA subsets involved in tumor proliferation, angiogenesis, and survival [103,104].

Tumor heterogeneity in humans impacts the immunoexpression of proteins in the mTOR pathway in PC, and the decrease in mTOR immunoexpression is considered a late event after tumor progression, being associated with worse clinical and oncological outcomes in patients [105]. These aspects are supported by the fact that the upregulation of this protein occurs in precursor lesions and the beginning of PC development. In contrast, human PC tends to exhibit negative expression of mTOR and 4E-BP1 in advanced cases with higher Gleason score, with lower protein levels of 4E-BP1 [103,105,106].

However, the immunoexpression of these proteins in the canine species seems to occur differently. According to Rivera-Calderón et al. [107], canine PC has high protein levels of mTOR and eIF4E when compared to normal prostate tissue, especially in samples with higher Gleason scores. Although not common, cases with GS ≥ 9 may show mutual overexpression for 4E-BP1 and eIF4E, as well as for their respective proteins, but only the highest gene and protein expression of the eIF4F translation initiation complex seems to be overexpressed in samples of metastatic tissue [107].

Unlike humans, no evidence of mutations in the mTOR kinase domain is observed in dogs, thus ruling out the involvement of this epigenetic change in prostate carcinogenesis in the canine species, requiring further investigation of this signaling pathway to better elucidate the differences involved between canine and human species [107]. Despite this, these findings reaffirm the action of this pathway in the encoding process of pro-oncogenic proteins acting in cell cycle advancement, cell survival, energy metabolism, angiogenesis, and metastatic potential [108].

### 7.3. Ki67 Cell Proliferation Markers and Epidermal Growth Factor

Involved in cell proliferation and tumor growth, Ki67 proteins and epidermal growth factor (EGF) are used as important predictive markers in patients with prostate cancer. In men, the highest Ki67 immunoexpression is seen in PC patients with a high Gleason score, more advanced tumor stage, and increased risk of death [109]. Furthermore, this marker is directly related to EGF, as Ki67+ epithelial cells also express high levels of phosphorylated epidermal growth factor receptor (pEGF-R) and human epidermal growth factor receptor 2 (HER2) [109].

In dogs, PC has a high proliferative rate and is associated with Ki67 immunoexpression, which is significantly up regulated compared to benign and pre-neoplastic lesions [96]. Similar results were also found by Fonseca-Alves et al. [110], who described a positive association between transcriptional levels of c-KIT with the number of Ki67-positive cells. According to the authors, no correlation is observed between Ki67 and the histological PC subtype, and the absence of clinical data makes it difficult to identify any correlation between survival time and prognosis using these markers [110].

As Ki67, HER1, and HER2, members of the epidermal growth factor receptor (HER) family are also up regulated in prostate cancer [111,112]. Reports have indicated a gain in the number of copies of the HER2 gene, which results in frequent overexpression of the protein not only in prostate tumors [113] but also in PIA [111]. Faleiro et al. [111] detected mRNA of the HER1 gene in all evaluated cases of canine PC although present in normal tissue and pre-neoplastic lesions. Thus, these results reaffirm the idea that these receptors are involved in carcinogenesis and tumor development in the canine prostate.

### 7.4. Oncogenes and Tumor Suppressor Genes

In healthy cells, proto-oncogenes act as important growth factors, cell signal transducers, and nuclear transcription factors. However, when these genes undergo mutations, they are called oncogenes and act as inducers of malignant phenotype in target cells, playing a fundamental role in carcinogenesis [114]. In contrast, tumor suppressor genes are crucially involved in repairing DNA damage, inhibiting cell division, inducing apoptosis, and suppressing metastases, that is, the loss of their function implies cancer development and progression [114].

Activation or overexpression of oncogenes triggers different biochemical responses, such as target molecule phosphorylation and induction of DNA transcription, often associated with several tumor suppressor genes [115,116]. Although more than 60 oncogenes are known, some are known to be involved in PC carcinogenesis, such as c-KIT [110], BRAF [117], C-MYC [118] and MDM2 [95]. Likewise, some tumor suppressor genes have also been investigated in PC, such as PTEN, NKX3.1, and TP53, which tend to be down-regulated [95,118].

From the tyrosine kinase receptors group, the stem cell factor receptor c-KIT belongs to a complex system of intracellular signal transduction pathways [119], involved in the progression of human prostate cancer, in the alteration of the bone microenvironment, resulting in migration from primary cancer cells to bones [120]. However, canine PC cells can induce cell proliferation mediated by autophosphorylation by exhibiting high transcriptional levels of c-KIT, but its expression in this neoplasm cannot be associated with the metastatic process in individuals of this species [110].

In addition to its role in regulating cell proliferation, c-KIT appears to be involved in regulating the survival and activation of mast cells, cells involved in tumor-microenvironment interaction in the canine PC [121]. In humans, normal prostate peripheral stromal cells have been associated with the development of PC through the c-KIT signaling pathway [122], reinforcing the findings by Fonseca-Alves et al. [110]. According to the authors, some canine PC samples also exhibit positive c-KIT immunostaining in stromal cells, reflecting the importance of the stroma in maintaining the tumor microenvironment.

High levels of mRNA and C-MYC proteins have been reported in PC patients, contributing to cell self-renewal, survival, and growth, as well as ribosomal metabolism and synthesis [123]. In dogs, Fonseca-Alves et al. [118] demonstrate that PC progression is influenced by higher transcriptional levels of C-MYC and down-regulation of NKX3.1, the same scenario observed for the levels of protein products of these genes. However, these regulatory mechanisms are believed to occur differently in dogs, as there is no hypermethylation of NKX3.1 in the prostate of these animals [118].

Furthermore, overexpression of C-MYC can antagonize AR target genes in men since both co-occupy a substantial number of binding sites and exhibit enhancer-like characteristics [124]. This antagonistic relationship can be explained by the fact that defects in the NKX3.1 gene, such as allelic loss, haploinsufficiency, attenuated expression, or decreased protein stability, represent established pathways in prostate tumorigenesis, with a close relationship with androgen resistance [125].

In addition to NKX3.1, two other important tumor suppressor genes involved in prostate carcinogenesis include TP53 and phosphatase and tensin homolog (PTEN). Responsible for inhibiting cell growth, increasing cell sensitivity to apoptosis, and repairing DNA damage, any mutations in these suppressor genes or changes in the expression of their respective proteins result in genetic instability, making them molecular landmarks for carcinogenesis [126,127]. Both are involved in a complex signaling network with the murine double minute gene-2 (MDM2), an oncoprotein involved in cell survival and neoplastic development [128].

PTEN protein is considered a p53 protector, as it modulates the MDM2 function in the cell cytoplasm. However, PTEN loss allows MDM2 translocation to the nucleus, which results in the inhibition of the p53 transcriptional activity [129,130,131]. In addition to AR, PTEN also presents negative regulation in canine PC samples, similar to what happened in cases of hormone-refractory prostate cancer in men, but with overexpression and amplification of MDM2, with marked nuclear immunostaining of its protein [95]. Thus, the down-regulation of PTEN in canine PC is also believed to be associated with loss of copy number on chromosome 26, the degree of cell differentiation, and, consequently, biologically aggressive neoplasms [95].

In contrast, Pagliarone et al. [131] reported moderate to severe p53 immunostaining in 69% of canine prostate cancer cells, particularly in those with a metastatic profile. According to the authors, the increased expression of the p53 protein in prostate cancer corresponds to defects in its corresponding gene, providing an apoptosis-resistant phenotype. In men, p53 mutation has also been observed in advanced and metastatic PC, but it is believed to be associated with the signaling pathway along CD24 [132], a cell surface coding protein involved in promoting immune escape, thus favoring tumor progression and metastatic capacity [133].

The PTEN down-regulation also occurs when there is protein overexpression of the signal transducer and activator of transcription (STAT3), mainly in solid tumors with a lower degree of differentiation [134]. Like MDM2, STAT3 is a transcription factor regulating tumor suppressor gene, which is physically and functionally connected to p63, both also involved in the regulation of normal stem cells and CSC [135]. Furthermore, expressed in hyperplastic canine prostate lesions, STAT3 acts in the regulation of malignant transformation and, therefore, can be used as a diagnostic marker for canine PC [134].

As for TP53, mutations in the BRAF gene have been found with significant frequency in urothelial carcinoma and canine PC, which allowed the inference that they originate in the urothelium in many cases of PC [136]. The protein encoded by BRAF is a component of the mitogen-activated protein kinase (MAPK)/extracellular signal-regulated kinase (ERK) signaling pathway, which acts in the regulation of cell proliferation, differentiation, and apoptosis, responding to extracellular stimuli (cytokines, growth factors, hormones, and environmental stressors) [137].

Considered uncommon in cases of prostate cancer in men, BRAF gene mutation was detected with high frequency in canine PC samples with high Gleason scores, with occurrence varying in 61–80% of the studied cases [117,136]. The analysis of this gene mutation was considered a highly specific method to assist in PC diagnosis when the cyto-histopathological examination is questionable since such alteration was not identified in normal prostate tissue or other prostatic disorders, such as hyperplasia, squamous metaplasia, prostatitis, and atrophy [117]. However, the occurrence of mutation of this gene in pre-neoplastic lesions such as PIA and PIN has not been investigated yet.

### 7.5. Cell Adhesion and Epithelial-Mesenchymal Transition

PC has high metastatic potential in men and dog, mainly to lymph nodes, lungs, liver, and bones [4,138,139]. The metastatic process is complex and represented by local invasion of blood and lymphatic vessels, as well as transport, extravasation, and colonization, with positive or negative regulation of several genes and their respective proteins, many of which are involved in cell differentiation [4] and previously discussed in this review.

**Figure 4:**
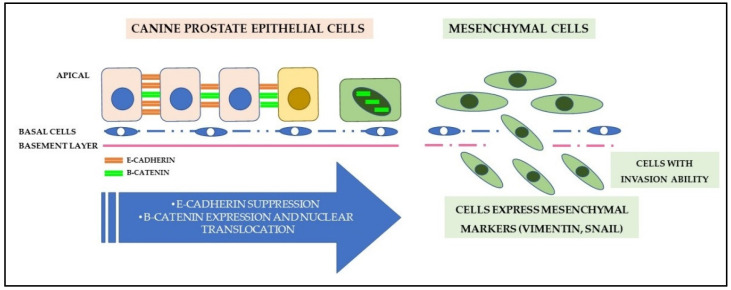
Loss of cell adhesion and epithelial-mesenchymal transition of canine PC. Canine prostate tumor cells overexpress β-catenin, which is accumulated in the cell nucleus, binding to transcription factors to activate gene expression. Concomitantly, there is suppression of E-cadherin, resulting in important structural changes, such as loss of adhesion of neoplastic cells. Following this process, especially in tumors with a lower degree of cell differentiation, tumor cells undergo immunophenotypic changes, expressing mesenchymal markers (vimentin and snail). Such mechanisms significantly influence the process of invasion, migration and metastasis of neoplastic cells.

Loss of cell adhesion and epithelial-mesenchymal transition (EMT) consist of events involved in the progression, invasion, and metastatic capacity of PC (Figure 4). These modifications are strongly associated with the WNT canonical signaling pathway [140]. The WNT family comprises a set of cysteine-rich lipoglycoproteins responsible for stem cell self-renewal and cell proliferation, migration, and differentiation [140]. WNT lipoglycoproteins bind to transmembrane receptors (frizzled receptors and low-density lipoprotein receptor) and activate the canonical pathway, promoting the stabilization and nuclear translocation of the β-catenin protein [141]. The action of β-catenin is influenced by the adenomatous polyposis coli protein (APC), a tumor suppressor [142], and E-cadherin [143].

The E-cadherin/β-catenin transmembrane complex is important for the maintenance of transmembrane epithelial adhesion in prostate cells [144], but, when signaled, the WNT pathway can induce the action of E-cadherin suppressors (Snail family), which results in negative regulation [145]. Consequently, the loss of E-cadherin expression will allow the dissociation and translocation of β-catenin from the cytoplasm to the nucleus, increasing the mobility of neoplastic cells, as well as their local invasion capacity, in addition to being used as an important marker of EMT during tumor progression and metastasis of human and canine PC [146,147,148].

The dysregulation of WNT signaling is considered a critical event in the carcinogenesis and progression of prostate cancer in humans, for which different molecular mechanisms responsible or involved in the aberrant activation of this pathway were evidenced. Among them, the upregulation of kinesin motor proteins [149], overexpression of proteins belonging to the epidermal growth factor family [150], epigenetic modifications [151], and dysregulation in the expression of micro-RNAs [152] and long non-coding RNAs [153,154]. Although not well elucidated in the canine species, understanding the signaling mechanisms of the WNT pathway will allow a better understanding of tumor biology and, consequently, advance the search for new therapeutic targets [155].

Changes in the expression of E-cadherin and β-catenin have been described in pre-neoplastic lesions and canine prostate tumors, being associated with higher cell proliferation rates [144,156]. Additionally, Kobayashi et al. [157] presented clarification about the involvement of the expression of these proteins in WNT signaling in cases of canine PC. According to the authors, there is overexpression of β-catenin, with loss of membranous marking and nuclear expression gain, especially in metastatic cases. Translocation of E-cadherin from the membrane to the cytoplasm, heterogeneous cytoplasmic labeling, and eventually nuclear, with loss of expression of the APC protein, are associated with these changes. However, there is no APC hypermethylation in canine PC, an important epigenetic change seen in human PC [157].

Canine patients with lower gene and protein expression of E-cadherin tend to have a shorter survival time and PC with a higher Gleason score. In contrast, cells positive for this protein are found in large quantities in metastatic foci [157,158]. The negative expression of E-cadherin in men is associated with several molecular and epigenetic events, such as somatic mutations [159] and DNA methylation [160]. However, only hypermethylation of the CDH1 promoter has been currently identified as an epigenetic alteration responsible for the dysregulation of E-cadherin in metastatic PC samples in dogs [158].

A component of E-cadherin-based adherens junctions in epithelial cells, Nectin-4 also appears to be involved in canine prostate tumorigenesis and metastatic potential [161]. In this context, Salda et al. [161] point out that the cytoplasmic expression of Nectin-4 is increased in cases of canine PC, regardless of the histological subtype, except for solid PC, which does not express this protein. In addition to cytoplasmic labeling, metastatic cells tend to exhibit strong membranous positivity associated with nuclear p63 labeling. These findings suggest that the loss of these adhesion molecules facilitates the migratory phase of tumor cells and their re-expression during the typical clustering mode in prostate tumor metastases, but studies with a higher number of evaluated cases are still needed [161].

Accompanied by the loss of epithelial adhesion, neoplastic cells undergo immunophenotypic changes characterized by the loss of epithelial markers and positive expression of mesenchymal markers. This process, called epithelial-mesenchymal transition, can be directly or indirectly regulated by molecular mechanisms, including growth factors, interleukins, and cytokines, allowing prostate cancer cells to migrate or metastasize to different organs [162]. This process is known in human PC and involved underlying molecular mechanisms such as the Smad3/Sox5/Twist1 [163] and Notch [164] pathways have been described.

Studies have shown that these changes also occur in canine PC, considering that, in many cases, neoplastic luminal cells express mesenchymal markers in association with changes in the expression of β-catenin [147] and E-cadherin [165]. Furthermore, vimentin expression is markedly higher in metastatic cells than in primary carcinomas [144,165]. Elshafae et al. [138] reinforced this immunophenotypic pattern when evaluating different metastatic canine PC cell lines with osteomimetic properties. The authors detected increased mRNA expression of mesenchymal markers, mainly vimentin, revealing a pattern of poorly differentiated cells.

The role of the gastrin-releasing peptide receptor (GRPr) was demonstrated in the search for a better understanding of the signaling pathways involved in the EMT status of canine PC [166]. A member of the G protein-coupled receptor superfamily, the main agonists for these receptors are functionally and structurally similar to bombesin (BBN), involved in tumor growth and differentiation in several solid neoplasms [167]. According to Elshafae et al. [166], primary canine PC has increased the expression of GRPr mRNA, and BBN increases in vitro cell proliferation and migration, as well as in vivo tumor growth and invasion. Moreover, there is the upregulation of mesenchymal markers, with significant changes in the morphology of neoplastic cells, which acquire a spindle cell phenotype [166].

### 7.6. Angiogenesis

Prostatic epithelial cells in dogs can produce and overexpress a significant amount of angiogenic factors. Patients with PC with a high Gleason score exhibit marked expression of vascular endothelial growth factor-A (VEGF-A) and its receptor (VEGFR-2), with a significant increase in microvascular density [168]. Although the increase in the number of blood vessels is strongly associated with tumors with a high Gleason score, Leis-Filho et al. [168] demonstrated that this correlation does not exist with increased expression of VEGF-A. However, the authors state that VEGFR-2 can be used as a potential prognostic marker, as its higher expression occurs in patients with a shorter survival time [168,169].

Absent in normal and hyperplastic prostates, PECAM-1 (platelet endothelial cell adhesion molecule-1), VEGF, and Tie-2 (receptor tyrosine protein kinase-2) proteins are expressed in canine PC, with basal and secretory cells exhibiting increased expression for FGF-2 (fibroblast growth factor-2) [170]. Although VEGF and FGF-2 act together to stimulate the recruitment of perivascular cells and formation of functional vasculature [171], FGF-2 is also associated with epithelial and stromal proliferation in different prostate lesions, with an increase in immunoexpression in cases of hyperplasia and metastatic PC, as it induces numerical chromosomal defects and higher DNA breakage [172,173].

The ability of canine PC to simultaneously express different factors involved in the formation of new blood vessels is notorious, resulting in higher tumor proliferation and angiogenesis, whether of a paracrine or autocrine nature [170]. However, despite being similar, it is still necessary to elucidate the mechanisms and pathways involved in the regulation of angiogenic factors in canine PC samples [170], considering that some transcriptional factors, such as EGR1 [174] and FOXA1 [175], as well as the overexpression of genes involved in tumor suppression, such as MDM2 [176], have been identified as important direct or indirect regulators of angiogenesis in human PC.

### 7.7. Tumor Microenvironment: Structural, Inflammatory, and Metabolic Aspects

Among the changes in the tumor microenvironment, alterations in the stroma and extracellular matrix (ECM) play a dynamic role in cancer progression, mainly characterized by the remodeling of the local environment resulting from biochemical and structural changes that denote a strong interaction between cellular and non-cellular components [177,178]. Significant structural and functional differences in ECM have been observed in human PC cell lines from pro-metastatic orthotopic tumors compared to the less aggressive phenotype, exhibiting a higher number of cancer-associated fibroblasts (CAFs) and type I collagen fibers [179].

Studies that investigate the correlation between changes in ECM and canine PC aggressiveness are still incipient in veterinary medicine. However, Rivera-Calderón et al. [180] characterized, for the first time, collagen fibers (I, III, IV) and elastin in ECM of normal and neoplastic prostates in the canine species. The results of the study do not indicate differences in the distribution and location of collagen and elastin, except in PC with a solid pattern, in which there is loss of the type IV collagen. Decreased collagen in PC has also been described in cases of humans with PC of different Gleason scores, suggesting that it is due to the high activity of metalloproteinases [181], proteolytic enzymes from ECM also overexpressed in canine PC [182]. Moreover, decreased type IV collagen is associated with higher metastatic potential, as its function is to provide structure, support, and resistance to traction, also acting as a regulator of chemotaxis and cell adhesion and migration [183].

CAFs are also involved in the energy metabolism of prostate cancer cells in men [178]. Malignant epithelium interacts with other components of the tumor microenvironment and, when corrupted, CAFs induce the Warburg effect, which indirectly or directly results in the production and secretion of important metabolic intermediaries involved in energy production [184]. Unlike dogs, studies in humans have shown some mechanisms and expression of markers involved in the modulation of PC energy metabolism [185,186]. One of the signaling pathways involved is related to the role of the STAT3 oncogene in the regulation of genes related to cell metabolism [187]. However, the correlation between STAT3 and PC energy metabolism has not yet been investigated although it is overexpressed in canine PC.

Despite the few studies in this field, canine PC and preneoplastic lesions (PIA and PIN) show higher expression of urokinase-type plasminogen activator receptor (uPAR) than normal and hyperplastic tissues [188]. The overexpression of this marker in the inflammatory and neoplastic microenvironments of the prostate leads to a disorder in the regulation not only of proteolytic activity, but also the modulation of energy metabolism and promotion of the basal membrane and ECM degradation, thus favoring angiogenesis, proliferation, survival, and migration of neoplastic cells to other sites [189].

The inflammatory process can also modify the tumor environment and the overexpression of cytokines involved in the expression of glial cell-derived neurotrophic factor (GDNF) and FGF-1 results in the proliferation of epithelial and glandular stromal cells [173]. In this sense, the presence of stromal inflammatory cells in benign and malignant human prostate tissue is a common finding on histopathological examination, which also occurs in the gland of the canine species [190]. Stromal lymphoplasmacytic infiltrate is frequent in canine PC and appears more pronounced in tumors with urothelial differentiation and castrated dogs [74]. Furthermore, the presence and constant stimulation of inflammation trigger oxidative stress and genomic damage, playing an important role not only in carcinogenesis and tumor progression [190] but in the survival of the affected patient, depending on the cell type involved [191,192].

Studies have reported immune cells in the human PC tumor microenvironment, mainly T lymphocytes, but a detailed subset of phenotyping and functional characterization has not been presented [193]. Neoplastic lesions of the prostate in dogs tend to exhibit a higher density of T and B cells when compared to normal and hyperplastic tissues. Importantly, T lymphocytes are the largest infiltrative population in canine PC and show a positive correlation with the survival rate of animals [194]. In contrast, undifferentiated histological subtypes have fewer lymphocytes, regardless of type [194]. However, the role of lymphocytes in the immunological modulation of the canine PC tumor microenvironment is still considered controversial and speculative despite being one of the focuses of immunotherapy [195].

Although little understood, mast cells are also identified as a critical component in the stroma of the tumor microenvironment. Mechanisms of mast cell recruitment in the tumor microenvironment were evidenced when using human PC cell lines, contributing to the neoformation of blood vessels [196]. This tumor-microenvironment interaction was also observed by Defourny et al. [121]. According to the authors, canine PC lesions have a high density of mast cells with periglandular/peritumoral distribution. However, unlike men, there is no positive correlation between tryptase, one of the serine proteases secreted by mast cells, and the higher density of microvessels in PC in dogs, requiring the investigation of other pro-angiogenic proteases [121].

Some inflammatory cells, mainly macrophages and neutrophils, produce leukotrienes, pro-inflammatory lipid mediators derived from the 5-lipoxygenase (5-LO) pathway of arachidonic acid metabolism, involved in different pathological conditions, such as cancer [197]. Overexpression of 5-LO is observed in human PC, with high concentrations of its metabolites in metastatic cases [198]. This protein also plays a causal role in the maintenance of stem cells through the regulation of Nanog and C-MYC [199]. However, the expression of this marker in dogs occurs similarly in PC, hyperplasia, and prostatitis, in addition to being found less frequently in metastatic cells [200]. Goodman et al. [200] attribute this difference to a probable modification in the transcription or translation of 5-LO, associated with the development of a metastatic phenotype. Nevertheless, these interpretations should be handled with caution, because it is overexpressed in other neoplasms of the genitourinary tract [201].

Also associated with arachidonic acid, cyclooxygenase (COX) exhibits strong basal immunoexpression in canine PC cells [202]. COX-2, a key enzyme in fatty acid metabolism, is up-regulated during inflammation and oncogenesis, being an important therapeutic target in prostate cancer [203,204]. Cytokines and interleukins can increase the immunoexpression of COX-2 in normal prostate cells, but not in canine PC cells, and the inflammatory infiltrate in the tumor tissue was associated with lower expression of this enzyme [202]. Although these findings indicate that the increase in COX-2 expression is not related to inflammation in PC, Rodrigues et al. [205] showed its higher expression in pre-neoplastic lesions, indicating cooperation in the tumorigenesis process of the prostate associated with TGF-β.

COX-2 expression results in increased cell proliferation rate, alteration in sex steroid metabolism, and synergistic action with hormonal changes [206,207]. For this reason, non-steroidal anti-inflammatory cyclooxygenase inhibitors have been used in association with other therapeutic methods to treat canine PC [208]. However, Packeiser et al. [209] showed COX-2 activity in canine PC cell lines, but no influence on the metabolic activity and cell proliferation capacity was observed when treated with meloxicam. The authors reported an increase in prostaglandins after exposure, but meloxicam did not influence this production in one of the tested lines, which also showed lower levels of COX-2. It was possible to conclude that differences in response to the use of NSAIDs suggest gene mutations in more aggressive and metastatic tumors, in spite of the controversial results [209].

## 8. MicroRNAs (miRs)

Recent research has investigated the expression and function of various microRNAs (miRs) as human PC-specific predictive markers [210,211,212]. miRs are small non-coding RNA molecules 19–25 nucleotides in length, responsible for regulating post-transcriptional gene expression by inhibiting the translation of mRNA into a protein so that a portion of the miRs is released into the circulation as exosomes [213,214]. Involved in physiological phenomena such as development, differentiation, proliferation, cell migration, and apoptosis, microRNA expression is customarily unregulated in cancer and may act as oncogenes or tumor suppressors [215,216].

Kobayashi et al. [217] used the real-time quantitative PCR technique (q-RT-PCR) to investigate, for the first time, the expression levels of 277 miRs in non-tumor canine prostate tissue and prostate adenocarcinoma, aiming to identify those associated with this type of cancer. Five miRs (miR-18a, 95, 221, 222 and 330) were up-regulated and 14 miRs (miR-127, 148a, 205, 299, 329b, 335, 376a, 376c, 379, 380, 381, 411, 487b and 495) were down-regulated, specifically in canine PC. Importantly, many of the identified miRs are related to cell cycle progression, regulation of androgen receptors, resistance to radiotherapy, invasion and metastasis, malignant transformation, and some are regulated by immuno-inflammatory molecules [217].

miRs also circulate in biological fluids such as blood and urine, thus enhancing their potential as a biomarker. In humans, circulating levels of miR-182-5p and miR-375-3p were significantly increased in patients with PC, being associated with a more advanced stage of the disease, especially in patients who developed metastasis [218]. Only a few miRs show consistent associations between the different performed studies, making researchers look for more robust diagnostic models using highly sensitive and specific combinations of miRs, regardless of the Gleason score and the clinical stage of the patient [219].

## 9. Prostate-Specific Antigen (PSA)

In humans, prostate-specific antigen (PSA) is considered one of the main screening markers for prostate lesions and diagnostic guidance [220]. However, it is not a specific marker for carcinomas, indicating only prostate damage. Therefore, its use in routine is still discussed, considering that it is limited as a specific diagnostic marker. In contrast, very low levels of this biomarker are expected in patients who underwent surgical treatment for PC. Moreover, PSA levels increase again in cases of tumor recurrence, a process called biochemical recurrence. In these cases, the diagnostic accuracy of this marker is high [221].

On the other hand, the use of biomarkers in dogs is controversial. Dogs lack the KLK3 gene, which encodes the PSA protein. Therefore, PSA is not a protein that can be detected in these animals. However, they present a homologous protein called canine prostatic specific esterase (CPSE), which is encoded by the KLK2 gene, a homolog of the KLK3 gene [222]. The homology between CPSE and PSA is 58% when considering the amino acid sequence. Therefore, it would be a homolog for the dog [222]. Recently, some studies have tried to associate the expression of CPSE with different prostate lesions. Holst et al. [223] evaluated CPSE concentrations in dogs according to prostate volume and found no association of this marker with prostate volume. However, the authors identified an increase in CPSE relative to the basal value of each animal during the follow-up, considering the basal values previously evaluated. In practice, it would not be a routine assessment to be performed, as there was no association with the patients’ diagnosis [223]. Moreover, although CPSE presents high concentrations in the seminal fluid, these values are unknown in the different prostate changes in dogs.

The anti-human PSA antibody in tissue samples has been used for the diagnosis of canine prostate cancer, but without validation. Anti-PSA monoclonal antibodies for human use do not usually react with canine tissue. However, there is a high homology between kallikreins and, therefore, there are chances of immunostaining of different kallikreins using the anti-PSA polyclonal antibody. Thus, the polyclonal anti-PSA antibody has been used to identify CPSE [72,165,224].

## 10. Future Directions

Many challenges need to be overcome in order to properly diagnose and treat canine PC, given that the diagnostic and therapeutic approaches to canine prostate disorders still face many limitations and hardly any evolution has occurred in recent years. Thus, the first initiative was published in 2019, suggesting the standardization of the terminology of prostate changes in dogs, including the different histological subtypes [53]. This classification, considering the proposed standardization of prostate lesions, should be applied and correlated with clinical factors of patients to assess their diagnostic and prognostic value. In the personal experience of these authors, in routine, many clinicians and veterinary surgeons do not request a histomorphological evaluation believing that it will not provide relevant information. Therefore, it is essential to associate the histological criteria, including classification, with the patients’ prognosis for a better approach.

Still regarding the diagnosis, a major problem related to PC is the definition of the origin of the tumor (luminal × urothelial). Canine PC is considered an aggressive disease and shows undifferentiated subtypes when the histomorphological analysis is performed [53]. The degree of undifferentiation makes the phenotypic approach to these cases difficult to identify the origin of these tumors. Uroplakin III is considered one of the main markers of cells with urothelial origin. In contrast, cytokeratins 8/18 are the main luminal markers [72]. However, these markers are more expressed in well-differentiated cells, losing their diagnostic value in undifferentiated ones. Since the tumor may have a luminal or urothelial origin, further studies characterizing new diagnostic markers are essential for the identification of these two entities and assessment of their isolated prognostic value.

An initiative that could be important for future studies is the creation of an international consortium, where veterinarians who diagnose or research PC can contribute to unique studies, including more patients, with relevant clinical information. The union of researchers from different regions may even allow the morphological, phenotypic, and molecular comparison of PC from castrated and non-castrated animals. Although canine PC is considered more frequent in castrated dogs [225] in countries such as the United States and Canada, a similar incidence in castrated and non-castrated animals has been reported in Brazil [33]. Thus, tumor characterization in castrated or non-castrated dogs can bring new diagnostic and therapeutic perspectives.

The understanding of canine tumors has taken a new direction with the advent of molecular biology, with many tumors being diagnosed through molecular testing. Canine PC was no different, and two large previous molecular studies were performed [4,226,227,228]. Genomic, transcriptomic, and proteomic studies involving canine PC may help to identify diagnostic and prognostic markers, as well as serum markers, particularly those involving samples of bladder tumors, as they have a urothelial origin. Thus, the comparative evaluation can help in the taxonomy of these neoplasms.

## 11. Final Considerations

PC is a neoplasm of high genomic complexity in humans and dogs, but with significant molecular similarities in these species. So far, important genes and signaling pathways have been identified and clarified in canine PC so that many molecular profiles can stratify the different tumor phenotypes exhibited by this neoplastic entity, especially those with high metastatic potential. However, it is still necessary to associate and evaluate these results together with important clinical data, such as the patient survival rate. Despite the questions still to be clarified, the discoveries reached so far allow us to infer about the adoption of future biomarkers for diagnosis and prognosis in the veterinary and human clinical-oncological routine. Furthermore, the identification of genes and molecular pathways important and necessary for PC carcinogenesis and progression should be investigated as potential therapeutic targets, since there are still no efficient and responsive therapeutic protocols in the canine species. Thus, the findings so far reinforce the idea of using the dog as an ideal experimental model for a better understanding of aggressive prostate cancer resistance to androgen therapy in men.
